# Impact of baseline adipose tissue characteristics on change in adipose tissue volume during a low calorie diet in people with obesity—results from the LION study

**DOI:** 10.1038/s41366-024-01568-6

**Published:** 2024-06-26

**Authors:** Daniela Junker, Mingming Wu, Anna Reik, Johannes Raspe, Selina Rupp, Jessie Han, Stella M. Näbauer, Meike Wiechert, Arun Somasundaram, Egon Burian, Birgit Waschulzik, Marcus R. Makowski, Hans Hauner, Christina Holzapfel, Dimitrios C. Karampinos

**Affiliations:** 1 Institute of Diagnostic and Interventional Radiology, School of Medicine and Health, Technical University of Munich, Munich, Germany; 2 Institute for Nutritional Medicine, School of Medicine and Health, Technical University of Munich, Munich, Germany; 3 Department of Diagnostic and Interventional Radiology, University Hospital Ulm, Ulm, Germany; 4 Institute of AI and Informatics in Medicine, School of Medicine and Health, Technical University of Munich, Munich, Germany; 5 Else Kroener-Fresenius-Center of Nutritional Medicine, School of Life Sciences, Technical University of Munich, Freising, Germany; 6 Department of Nutritional, Food and Consumer Sciences, Fulda University of Applied Sciences, Fulda, Germany; 7 Munich Institute of Biomedical Engineering, Technical University of Munich, Garching, Germany; 8 Munich Data Science Institute, Technical University of Munich, Garching, Germany

**Keywords:** Obesity, Obesity, Anatomy, Medical research

## Abstract

**Background/Objectives:**

Weight loss outcomes vary individually. Magnetic resonance imaging (MRI)-based evaluation of adipose tissue (AT) might help to identify AT characteristics that predict AT loss. This study aimed to assess the impact of an 8-week low-calorie diet (LCD) on different AT depots and to identify predictors of short-term AT loss using MRI in adults with obesity.

**Methods:**

Eighty-one adults with obesity (mean BMI 34.08 ± 2.75 kg/m², mean age 46.3 ± 10.97 years, 49 females) prospectively underwent baseline MRI (liver dome to femoral head) and anthropometric measurements (BMI, waist-to-hip-ratio, body fat), followed by a post-LCD-examination. Visceral and subcutaneous AT (VAT and SAT) volumes and AT fat fraction were extracted from the MRI data. Apparent lipid volumes based on MRI were calculated as approximation for the lipid contained in the AT. SAT and VAT volumes were subdivided into equidistant thirds along the craniocaudal axis and normalized by length of the segmentation. *T*-tests compared baseline and follow-up measurements and sex differences. Effect sizes on subdivided AT volumes were compared. Spearman Rank correlation explored associations between baseline parameters and AT loss. Multiple regression analysis identified baseline predictors for AT loss.

**Results:**

Following the LCD, participants exhibited significant weight loss (11.61 ± 3.07 kg, *p* < 0.01) and reductions in all MRI-based AT parameters (*p* < 0.01). Absolute SAT loss exceeded VAT loss, while relative apparent lipid loss was higher in VAT (both *p* < 0.01). The lower abdominopelvic third showed the most significant SAT and VAT reduction. The predictor of most AT and apparent lipid losses was the normalized baseline SAT volume in the lower abdominopelvic third, with smaller volumes favoring greater AT loss (*p* < 0.01 for SAT and VAT loss and SAT apparent lipid volume loss).

**Conclusions:**

The LCD primarily reduces lower abdominopelvic SAT and VAT. Furthermore, lower abdominopelvic SAT volume was detected as a potential predictor for short-term AT loss in persons with obesity.

## Introduction

One strategy to prevent obesity-related comorbidities such as diabetes, cardiovascular disease, and certain cancers is a lifestyle intervention aiming at weight loss, ideally by reducing excess adipose tissue (AT) and ectopic fat [1]. The various fat depots have distinct metabolic profiles due to substantial functional differences [2]. Weight loss is also accompanied by loss of organ- and muscle mass; thus, the success of a weight loss intervention is best assessed by evaluating changes in the AT.

Outcomes of a short-term weight-reducing intervention have a significant impact on long-term weight maintenance success [3–5]. This short-term effect, in turn, varies substantially between persons, with varying loss of AT in the different AT depots. The ability to accomplish short-term weight loss is dependent on many factors, including adherence to the intervention [6], insulin resistance [7, 8], genetics and epigenetics [9, 10], gut microbiota [11], sleeping habits [12, 13] or basal metabolic rate [14, 15]. Age and sex are also factors often discussed, but findings from several studies are inconsistent [16].

The distribution of AT is also relevant for the success of a weight loss intervention. People with abdominal obesity and thus more visceral adipose tissue (VAT) benefit more from weight loss than those with gluteal-femoral obesity, who tend to have more subcutaneous adipose tissue (SAT) [17]. Such AT characteristics might be predictors for an intervention’s success but are mainly assessed by anthropometric measurements [16, 18, 19]. A few studies used imaging techniques and found that increased VAT, VAT/SAT ratio, or total AT at baseline are associated with greater weight- and VAT loss [20–25]. However, most studies performing a weight loss intervention while using imaging methods focused on the changes in AT instead of evaluating the association between baseline AT characteristics and the outcome of the intervention [8, 26–29]. Additionally, studies are limited by small sample sizes [23], by investigating single AT depots [30], or by using single-slice measurements leading to cross-sectional areas instead of measuring volumes for AT quantification [20–22, 24, 25, 31].

In general, methods that can assess the abdominopelvic fat content and distribution range from simple anthropometric measures to more complex 3D imaging technologies, such as Computed Tomography (CT) or Magnetic Resonance Imaging (MRI). These methods highly differ regarding accuracy, precision, and the amount of information gathered. 3D imaging technologies offer the highest precision and amount of collected data. MRI is the preferred method for AT imaging in healthy individuals since it does not require ionizing radiation. Single-slice measurements have poorly predicted VAT and SAT changes during weight loss [32]. In groups of people of different ages and sexes, more accurate results are achieved by measuring AT volumes in a volumetric approach [33]. Examining the whole abdomen and pelvis also allows assessment of the regional AT distribution in the craniocaudal axis. The current gold standard of MRI methods for spatially resolved tissue fat quantification are Dixon-based chemical shift encoding-based fat quantification techniques [34–37]. After considering multiple confounding factors, these techniques measure a tissue’s proton density fat fraction (PDFF) [38], offering a method to further characterize AT phenotypes.

The present study aimed to (1) assess AT and apparent lipid volume changes as meaningful parameters for the success of a weight loss intervention using an MRI-based approach and to (2) evaluate baseline parameters concerning their correlation with and their prognostic value for those changes. To achieve this, data from the lifestyle intervention (LION) study in people with obesity undergoing an 8-week formula-based weight loss intervention [39] was analyzed.

## Study cohort and methods

### Study design and participants

Between October 2019 and October 2021, a subgroup of 127 persons with obesity (73 females, mean age 45 years, BMI 30.0–39.9 kg/m^2^) were recruited from the LION study [39] to undergo an MRI examination of the abdomen and pelvis on a 3T scanner. Of those, 81 participants (49 females, mean age 46.3 years) completed a follow-up MRI scan after an 8-week formula-based low-calorie diet (LCD) of 800 kcal with an optional additional daily intake of 200 g non-starchy vegetables. The analysis presented in the following is based on the data obtained from the 81 participants who completed the study.

The study protocol and procedures were approved by the ethical committee of the School of Medicine and Health of the Technical University of Munich, Germany (Project Number 69/19S; ClinicalTrials.gov Identifier: NCT04023942). Written informed consent was obtained from all participants. Inclusion and exclusion criteria were defined as described elsewhere [39]; for the MRI examinations, additional exclusion criteria were standard MRI contraindications.

### Anthropometric measurements

Waist and hip circumferences were measured by trained study staff according to standard operating procedures [39]. Waist-to-hip ratio (WHR) was calculated as the ratio between waist- and hip circumference.

Weight was assessed in a fasted state in light clothing without shoes and with an emptied bladder using a body composition scale (BC-418MA, Tanita Europe B.V., Netherlands) for bioimpedance analysis (BIA). Since the participants were allowed to wear light clothing, one kilogram was deducted for the assessment. Weight (kg) and body fat (%, kg) were recorded. Height was measured in a standing position without shoes using a stadiometer (Seca 214, Seca, Hamburg, Germany). BMI was calculated as the quotient of weight in kilograms and height in meter squared (kg/m²) [39]. Participants underwent baseline and follow-up appointments at the Institute for Nutritional Medicine.

### MRI measurements

The MRI examinations of the abdomen and pelvis were performed on a 3T MR scanner (Ingenia Elition X, Philips Healthcare, Best, The Netherlands; software release 5.6). Participants were placed in supine position, head first, and a 16-channel torso coil and the build-in-table 12-channel posterior coil were used. For PDFF and volume measurements of AT, a 6-echo multi-echo gradient echo sequence with bipolar gradients was used in four stacks, covering the abdomen and pelvis from the liver dome to the center of the femoral heads. Each stack was acquired during a breath-hold scan of 10.3 seconds (see sequence parameters in Table S1). The mean time interval between the baseline clinical assessment (e.g., anthropometric measurements) at the Institute for Nutritional Medicine [39] and the baseline MRI scan was 8 days (range, 0–43 days). For the follow-up measurements after the LCD, the time interval was kept to a minimum with a mean of 1.7 days (range, 0–21 days).

#### PDFF mapping

PDFF, the state-of-the-art chemical shift encoding-based fat quantification technique [38], is defined as the proportion of mobile proton density in fat tissue attributable to fat. PDFF maps were generated using the vendor´s online complex-based fat quantification algorithm (Philips mDIXON Quant package, for scan parameters see Supplementary Table S1), accounting for the presence of multiple fat peaks, a single T2* correction, and phase errors [40].

#### Adipose tissue segmentation

VAT and SAT were segmented using a deep learning-based automated segmentation pipeline after [41, 42]. The segmented region extended from the liver dome to the middle of the femoral heads. For details and code availability, see [43, 44]. VAT and SAT volumes and mean AT PDFF values (%) were extracted. To investigate regional variations of VAT and SAT distribution in the craniocaudal axis, the segmentations were subdivided into equidistant thirds, starting at the liver dome and ending at the middle of the femoral head. A Python algorithm (Version 3.8.0, Python Software Foundation, Beaverton, USA) was established for these calculations. This resulted in SAT and VAT subvolumes for the segmented abdominopelvic region’s upper, middle, and lower third. The middle third included the periumbilical abdominal fat, and the lower third contained the pelvic and gluteal fat down to the hip joint. The lipid volume was calculated as ${{\rm{PDFF}}}\times {{\rm{Volume}}}$ for both AT compartments, thereby not taking into account MR-invisible components [45], thus the term “apparent lipid volume” is employed hereinafter. Absolute loss of total AT and apparent lipid volume was calculated as ${{{\rm{Volume}}}}_{{{\rm{follow}}}-{{\rm{up}}}}-\,{{{\rm{Volume}}}}_{{{\rm{baseline}}}}$ and expressed in L. Relative AT and apparent lipid volume losses were calculated as $({{{\rm{Volume}}}}_{{{\rm{follow}}}-{{\rm{up}}}}-{{{\rm{Volume}}}}_{{{\rm{baseline}}}})/{{{\rm{Volume}}}}_{{{\rm{baseline}}}}$ and expressed in %. Note that this method of volume loss calculation may result in negative values. VAT/SAT ratio was calculated as $\frac{{\rm{Volume}}_{{\rm{VAT}}}}{{\rm{Volume}}_{{\rm{SAT}}}}$ in the segmented region. Normalization of AT volumes (expressed in L/cm) was performed using the length of the segmented region in cm: $\frac{{{\rm{Volume}}}}{{{\rm{Length}}}}$. This was done in order to account for different physiques, as the height of the participants did not necessarily correlate with the length of the abdominopelvic region.

### Statistical analysis

Statistical analysis was performed using MedCalc Statistical Software (version 20.118; MedCalc Software bvba, Ostend, Belgium; https://www.medcalc.org; 2022). Normal distribution of data was verified using cumulative frequency distribution plots. Data is expressed as mean ± standard deviation, if not otherwise denoted. Differences between baseline and follow-up measurements were tested by means of the paired samples *t*-test. Sex differences and differences between AT depots were evaluated using the independent samples *t*-test. In order to compare the effect size of the intervention on the normalized AT volumes in each subvolume (upper, middle, lower third), standardized mean difference (SMD, Cohen’s *d*) was calculated. SMD can be interpreted based on [46], with an absolute value of >**│**0.2**│** representing a small effect, >**│**0.5**│** representing a medium effect, and >**│**0.8**│** representing a large effect. Due to the typical distribution patterns of AT in males and females, separate SMD analyses for males and females were performed. Correlation analyses were carried out using Pearson correlation. To determine which baseline parameter had the strongest effect on each change in AT (relative total volume loss Δ AT_TV_ and relative apparent lipid volume loss Δ AT_LV_ of SAT and VAT, respectively), stepwise multiple linear regression analyses were performed, including the five strongest associated parameters according to the Pearson correlation coefficient, and with the addition of age and sex as covariates. All analyses were conducted using two-tailed tests with a significance level of 5%, and no correction was made for multiple testing because of the explorative character of the study.

## Results

### Characteristics of the study cohort and changes following the LCD

The population characteristics at baseline and follow-up are shown in Table 1. Characteristics stratified by sex are presented in the Supplementary Material (Tables S2–S4).

**Table 1:** Characteristics of the study population (*n* = 81) at baseline and follow-up.

	Parameter	Baseline	Follow-Up	Difference baseline vs. follow-up
Characteristics & anthropometry	Females	49 (60.49%)	49 (60.49%)	–^a^
	Age, years	46.30 (10.97, 21–65)**	46.48 (10.95, 21–66)**	–^a^
	Height, m	1.72 (10.24, 1.49–1.98)**	1.72 (10.24, 1.49–1.98)**	–^a^
	Weight, kg	101.56 (15.17, 71.30–149.80)**	89.95 (13.76, 63.40–132.20)**	*p* < 0.01
	BMI, kg/m^2^	34.08 (2.75, 30.20–39.70)	30.19 (2.62, 26.10–36.00)	*p* < 0.01
	Waist circumference, cm	105.75 (11.54, 76–135.20)**	96.30 (10.21, 77.70–119.20)**	*p* < 0.01
	Hip circumference, cm	118.00 (8.07, 101.00–136.00)	110.37 (7.20, 97.30–128.40)	*p* < 0.01
	WHR	0.90 (0.09, 0.72–1.12)**	0.87 (0.08, 0.71–1.05)**	*p* < 0.01
	Body fat, %	38.61 (7.05, 22.80–52.30)**	34.53 (7.98, 18.90–51.00)**	*p* < 0.01
SAT	PDFF, %	90.39 (1.33, 86.18–92.61)**	88.43 (2.38, 80.61–92.20)**	*p* < 0.01
	Total volume, L	15.55 (3.96, 7.47–24.93)	12.31 (3.62, 5.37–20.27)	*p* < 0.01
	Upper third	4.03 (1.18, 1.43–6.89)	3.17 (1.10, 1.31–5.92)*	*p* < 0.01
	Middle third	5.25 (1.51, 2.49–8.98)	4.24 (1.35, 1.95–7.22)	*p* < 0.01
	Lower third	6.27 (1.60, 2.87–10.48)	4.90 (1.48, 1.86–8.40)*	*p* < 0.01
	Normalized volume^b^, L/cm	0.35 (0.09, 0.18–0.60)**	0.29 (0.08, 0.13–0.51)**	*p* < 0.01
	Upper third	0.27 (0.08, 0.11–0.53)**	0.22 (0.08, 0.09–0.45)**	*p* < 0.01
	Middle third	0.35 (0.10, 0.18–0.58)	0.29 (0.09, 0.13–0.53)	*p* < 0.01
	Lower third	0.42 (0.10, 0.20–0.68)**	0.34 (0.10, 0.12–0.59)**	*p* < 0.01
	Apparent lipid volume, L	14.09 (3.69, 6.44–22.77)	10.95 (3.39, 4.38–18.52)	*p* < 0.01
	Normalized apparent lipid volume^b^, L/cm	0.32 (0.08, 0.15–0.55)**	0.25 (0.08, 0.10–0.46)**	*p* < 0.01
VAT	PDFF, %	78.85 (4.10, 67.43–85.66)**	74.67 (4.83, 62.18–81.94)**	*p* < 0.01
	Total volume, L	5.70 (2.32, 1.91–12.93)**	4.46 (1.85, 1.56–9.81)**	*p* < 0.01
	Upper third	1.12 (0.64, 0.25–3.41)**	0.97 (0.58, 10.12–2.98)**	*p* < 0.01
	Middle third	2.92 (1.29, 0.79–6.75)**	2.22 (0.99, 0.65–4.96)**	*p* < 0.01
	Lower third	1.66 (0.57, 0.70–3.97)**	1.27 (0.42, 0.60–2.44)*	*p* < 0.01
	Normalized volume^b^, L/cm	0.13 (0.05, 0.04–0.28)**	0.10 (0.04, 0.04–0.22)**	*p* < 0.01
	Upper third	0.07 (0.04, 0.02–0.20)**	0.07 (0.04, 0.01–0.17)**	*p* < 0.01
	Middle third	0.20 (0.08, 0.05–0.44)**	0.15 (0.06, 0.05–0.33)**	*p* < 0.01
	Lower third	0.11 (0.04, 0.05–0.26)*	0.09 (0.03, 0.05–0.17)	*p* < 0.01
	Apparent lipid volume, L	4.57 (2.04, 1.29–11.08)**	3.40 (1.57, 0.97–8.04)**	*p* < 0.01
	Normalized apparent lipid volume^b^, L/cm	0.10 (0.04, 0.03–0.24)**	0.08 (0.03, 0.02–0.18)**	*p* < 0.01
	VAT/SAT ratio	0.39 (0.21, 0.12–1.15)**	0.39 (0.20, 0.13–1.07)**	*p* = 0.61

The significance level for sex differences is marked as * for *p* < 0.05 and ** for *p* < 0.01. Data are shown as mean (SD, range) or as *n* (%).

*MRI* magnetic resonance imaging, *SAT* subcutaneous adipose tissue, *VAT* visceral adipose tissue, *PDFF* proton density fat fraction, *BMI* body mass index, *SD* standard deviation, *WHR* waist-to-hip ratio.

^a^Not changing with intervention.

^b^Normalized by the length of the abdominopelvic region in cm.

After the LCD intervention, a mean weight loss of −11.61 ± 3.07 kg, a mean BMI decrease of −3.89 ± 0.88 kg/m², and a BIA-based body fat reduction of −4.08 ± 2.04% was achieved (Table 1). The imaging data revealed an absolute loss of SAT total volume (Δ SAT_TV_) of −3.24 ± 1.07 L and an absolute loss of SAT apparent lipid volume (Δ SAT_LV_) of −3.14 ± 1.03 L. Absolute loss of VAT total volume (Δ VAT_TV_) was −1.24 ± 0.66 L, and of VAT apparent lipid volume (Δ VAT_LV_) −1.17 ± 0.64 L (Table 1). Relative to baseline, loss of SAT total volume (Δ SAT_TV_) was −21.46 ± 6.73% (Δ SAT_LV_ −23.10 ± 7.44%), and Δ VAT_TV_ −21.79 ± 6.99% (Δ VAT_LV_ −25.83 ± 8.09%) (Table 1). The Δ SAT_TV_ (L) was significantly higher than Δ VAT_TV_ (L) (*p* < 0.01), while the Δ SAT_TV_ (%) and Δ VAT_TV_ (%) were not significantly different (Table 1). However, there was a significantly higher Δ VAT_LV_ (%) compared to Δ SAT_LV_ (%) (*p* < 0.01) (Fig. S1). The PDFF decreased both in SAT and in VAT, with a stronger decrease in VAT (*p* < 0.01).

The levels of significance of the differences between males and females at baseline and follow-up are marked by an asterisk in Table 1, and the corresponding numbers separated by sex are shown in Tables S2–S4. For males, Δ SAT_TV_ was −3.50 ± 1.23 L (Δ SAT_LV_ −3.41 ± 1.18 L) and Δ VAT_TV_ was −1.76 ± 0.68 L (Δ VAT_LV_ −1.68 ± 0.67 L) (Table S3). For females, Δ SAT_TV_ was −3.07 ± 0.93 L (Δ SAT_LV_ −2.96 ± 0.88 L) and Δ VAT_TV_ was −0.91 ± 0.37 L (Δ VAT_LV_ −0.84 ± 0.35 L) (Table S4). Relative to baseline, men showed a loss of −24.08 ± 7.25% SAT_TV_ (−26.27 ± 8.14% SAT_LV_) and −23.75 ± 7.24% VAT_TV_ (−27.95 ± 8.62% VAT_LV_) (Table S3), while women showed a loss of −19.75 ± 5.82% SAT_TV_ (−21.03 ± 6.20% SAT_LV_) and −20.50 ± 6.58% VAT_TV_ (−24.45 ± 7.50% VAT_LV_) (Table S4). The absolute volume loss of both total volume and apparent lipid volume was significantly higher in SAT compared to VAT (*p* < 0.01) in males and females. The sex-specific results for the relative AT- and apparent lipid volume losses are shown in Fig. S2.

When dividing SAT and VAT into equidistant thirds from the liver dome down to the middle of the femoral head, a significant change in all subvolumes for both SAT and VAT was observed (Table 1). However, the strongest effects were observed for both SAT and VAT in the lower third, with a large effect for SAT (0.81) and a medium effect for VAT (0.71). The evaluation in females revealed a large effect for the lower third SAT (0.89), while in males, large effects were seen in lower SAT and middle and lower VAT (0.81, 0.85 and 0.86, respectively; see Table 2).

**Table 2:** Standardized mean differences (SMD) for comparison of normalized SAT and VAT subvolumes (L/cm) before and after weight loss intervention (upper, middle, and lower third of the segmented region liver dome to femoral head) in the complete cohort and in males and females.

	Compartment	Subvolume	SMD	95% CI
All (*n* = 81)	SAT, L/cm	Upper third	−0.68	−0.81 to −0.54
		Middle third	−0.65	−0.77 to −0.55
		Lower third	**−0.81**	−0.95 to −0.66
	VAT, L/cm	Upper third	−0.22	−0.33 to −0.12
		Middle third	−0.59	−0.68 to −0.49
		Lower third	−0.71	−0.86 to −0.55
Females (*n* = 49)	SAT, L/cm	Upper third	−0.68	−0.88 to −0.49
		Middle third	−0.73	−0.93 to −0.54
		Lower third	**−0.89**	−1.13 to −0.65
	VAT, L/cm	Upper third	−0.22	−0.43 to −0.02
		Middle third	−0.62	−0.76 to −0.49
		Lower third	−0.62	−0.82 to −0.42
Males (*n* = 32)	SAT, L/cm	Upper third	−0.78	−0.95 to −0.61
		Middle third	−0.58	−0.68 to −0.48
		Lower third	**−0.81**	−1.02 to −0.63
	VAT, L/cm	Upper third	−0.36	−0.54 to −0.17
		Middle third	**−0.85**	−1.06 to −0.64
		Lower third	**−0.86**	−1.12 to −0.62

SMD > │0.8│ based on standardized mean differences represent a large effect and are printed in bold.

*CI* confidence interval, *SAT* subcutaneous adipose tissue, *SMD* standardized mean differences, *VAT* visceral adipose tissue.

### Association analyses

**Fig. 1:**
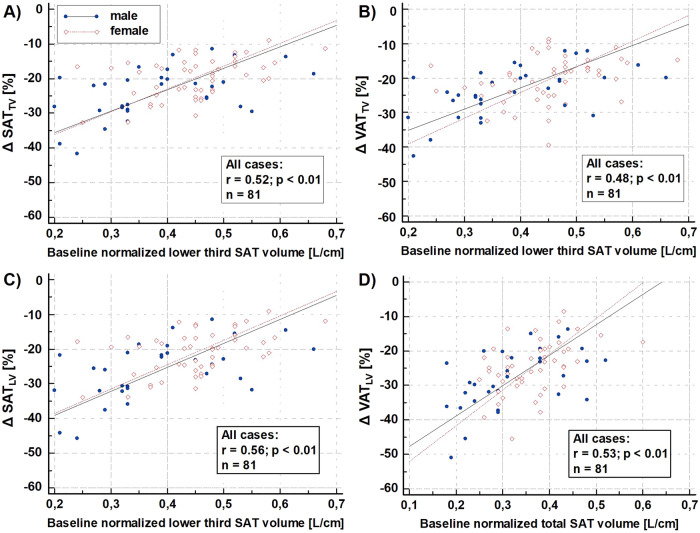
Scatter plots depicting correlations between relative AT and AT apparent lipid volume losses and respective baseline parameters. Blue dots represent male cases, open red diamonds represent female cases. **A** Correlation between ∆ SAT_TV_ (%) and baseline normalized lower third SAT volume. **B** Correlation between ∆ VAT_TV_ (%) and baseline normalized lower third SAT volume. **C** Correlation between ∆ SAT_L__V_ (%) and baseline normalized lower third SAT volume. **D** Correlation between ∆ VAT_TV_ (%) and baseline normalized total SAT volume. SAT subcutaneous adipose tissue, VAT visceral adipose tissue, ∆ SAT_TV_ (%) relative loss of SAT total volume, ∆ SAT_LV_ (%) relative loss of SAT apparent lipid volume, ∆ VAT_TV_ (%) relative loss of VAT total volume, ∆ VAT_LV_ (%) relative loss of VAT apparent lipid volume.

The Δ SAT_TV_ (%) correlated the strongest with the normalized baseline SAT volume (L/cm) in the lower third, with a higher loss (indicated by a negative number) being associated with smaller baseline volumes (*r* = 0.52, *p* < 0.01, Fig. 1). Other strong correlations of Δ SAT_TV_ (%) were found with normalized total SAT volume (L/cm), normalized SAT apparent lipid volume (L/cm), and BIA-based body fat (%) at baseline (*r* = 0.47, *r* = 0.47, *r* = 0.49, respectively; *p* < 0.01) (Table 3). Δ SAT_LV_ (%) showed associations similar to those of the aforementioned Δ SAT_TV_ (%) (see Table 3 and Fig. 1).

**Table 3:** Pearson correlation coefficients for VAT and SAT adipose tissue and apparent lipid volume loss with anthropometric and imaging parameters at baseline.

Parameters at baseline		∆ SAT_TV_ (%)	∆ SAT_LV_ (%)	∆ VAT_TV_ (%)	∆ VAT_LV_ (%)
Anthropometry	BMI, kg/m²	0.32**	0.33**	0.29**	0.34**
	Body fat, %	0.49**	0.53**	0.41**	0.42**
	Waist circumference, cm	0.09	0.10	0.19	0.23*
	Hip circumference, cm	0.36**	0.39**	0.28*	0.32**
	WHR	−0.15	−0.16	0.003	0.03
SAT	PDFF, %	0.33**	0.37**	0.39**	0.43**
	Normalized volume^a^, L/cm				
	• Total	0.47**	0.52**	0.48**	0.53**
	• Upper	0.39**	0.43**	0.43**	0.50**
	• Middle	0.36**	0.40**	0.39**	0.43**
	• Lower	0.52**	0.56**	0.48**	0.52**
	Normalized apparent lipid volume^a^, L/cm	0.47**	0.51**	0.47**	0.53**
VAT	PDFF, %	0.05	0.04	0.09	0.14
	Normalized volume^a^, L/cm				
	• Total	0.04	0.03	0.02	0.07
	• Upper	−0.02	−0.04	−0.08	−0.03
	• Middle	0.001	−0.01	0.01	0.06
	• Lower	0.20	0.21	0.18	0.24*
	Normalized apparent lipid volume^a^, L/cm	0.05	0.04	0.03	0.08
	VAT/SAT ratio	−0.24*	−0.28*	−0.25*	−0.24*

Significance is marked as * for *p* < 0.05 and ** for *p* < 0.01.

*SAT* subcutaneous adipose tissue, *VAT* visceral adipose tissue, *WHR* waist-to-hip ratio, *∆ SAT*_*TV*_ *(%)* relative loss of SAT total volume, *∆ SAT*_*LV*_ *(%)* relative loss of SAT apparent lipid volume, *∆ VAT*_*TV*_ *(%)* relative loss of VAT total volume, *∆ VAT*_*LV*_ *(%)* relative loss of VAT apparent lipid volume.

^a^Normalized by the length of the abdominopelvic region in cm.

The Δ VAT_TV_ (%) correlated best with normalized SAT volume in the lower third and normalized total SAT volume at baseline (both *r* = 0.48, *p* < 0.01). Other correlations were found with normalized SAT apparent lipid volume, normalized SAT volume in the upper third, and BIA-based body fat (%) at baseline (*r* = 0.47, *r* = 0.43, *r* = 0.41, respectively; *p* < 0.01 for all). Δ VAT_LV_ (%) correlated best with normalized total SAT volume, normalized SAT apparent lipid volume, and normalized SAT volume in the lower as well as the upper third at baseline (*r* = 0.53, *r* = 0.53, *r* = 0.52 and *r* = 0.50, respectively; *p* < 0.01) (Table 3 and Fig. 1). Notably, baseline SAT PDFF correlated with all AT and apparent lipid losses (each *p* < 0.01), while baseline VAT PDFF did not show any correlations (Table 3). The correlation analyses were also performed in males and females separately, as shown in Supplementary Tables S5 and S6.

Correlation analyses of weight loss and BMI reduction (both relative to baseline) with baseline MRI and anthropometric parameters revealed no correlations (data not shown). When males and females were considered separately, no correlation was found in males. In females, both relative weight- and BMI loss correlated with baseline body fat (%) (*r* = 0.38 for weight loss and *r* = 0.37 for BMI loss, both *p* < 0.01), waist circumference (*r* = 0.3, *p* = 0.04 for both), and SAT volume in the lower third (total SAT: *r* = 0.32, *p* = 0.03 for weight loss and *r* = 0.32, *p* = 0.02 for BMI loss; normalized SAT: *r* = 0.32, *p* = 0.03 for weight loss and *r* = 0.32, *p* = 0.02 for BMI loss), with smaller baseline values always favoring greater losses.

### Multiple regression models

**Fig. 2:**
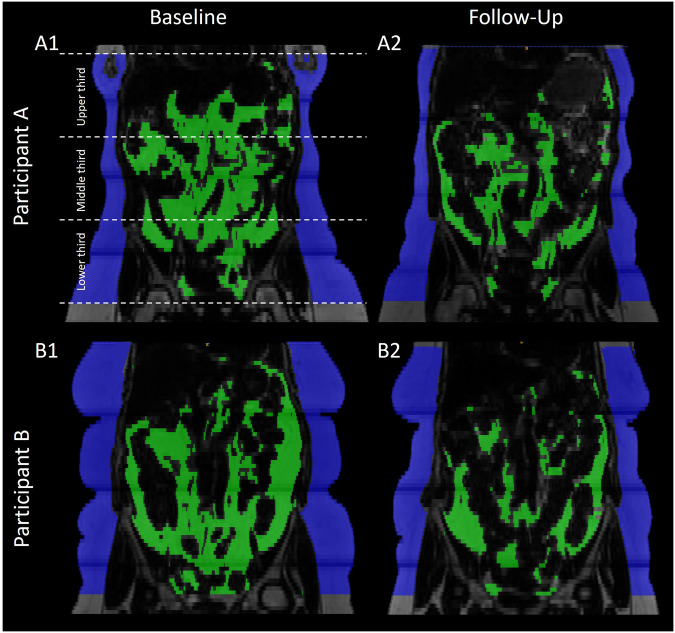
Segmented coronal fat images of two female participants at baseline and follow-up showing less VAT loss in a woman with more SAT in the lower third at baseline (participant A) compared to more VAT loss in a woman with fewer SAT in the lower third at baseline (participant B). VAT is marked in green, and SAT is marked in blue. The upper left image shows the concept of division into equidistant thirds (upper, middle, lower third). Participant A (female, 49 years, baseline BMI 32.1 kg/m²) with a SAT distribution with emphasis in the lower third (0.43 L/cm) lost 12.62% VAT after the 8-week calorie restriction. Participant B (female, 56 years, baseline BMI 32.2 kg/m²) with a more balanced SAT distribution with less SAT in the lower third (0.39 L/cm) lost 31.45% VAT after the 8-week calorie restriction. The biggest change in SAT is visible in the lower third of the segmented region in both participants. SAT (blue) subcutaneous adipose tissue, VAT (green) visceral adipose tissue.

Multiple linear regression models for the prediction of AT and apparent lipid volume losses (indicated by a negative number) showed that the normalized SAT volume in the lower third and body fat % at baseline are the predictors for Δ SAT_TV_ (%) (*b* = 0.24, *p* < 0.01 and *b* = 0.003, *p* = 0.02), with less SAT at baseline being beneficial for SAT loss. The same was true for Δ SAT_LV_ (%) (*b* = 0.28, *p* < 0.01 and *b* = 0.003, *p* = 0.01). For Δ VAT_TV_ (%), the predictor was normalized SAT volume in the lower third (*b* = 0.32, *p* < 0.01), and for Δ VAT_LV_ (%), the predictors were normalized total SAT volume and normalized SAT volume in the middle third (*b* = 1.03, *p* < 0.01 and *b* = −0.49, *p* = 0.04, respectively). Overall, a smaller SAT volume in the lower third at baseline was advantageous for SAT- and VAT loss (Table 4). The impact of SAT distribution on VAT loss is visible in Fig. 2, showing a woman with more baseline SAT in the lower third exhibiting smaller VAT loss compared to a woman with less SAT in the lower third at baseline exhibiting greater VAT loss. The reported multiple regression analyses were not performed separated by sex as the number of independent variables in the model (*n* = 7) was too large for the respective groups (*n* = 32 and *n* = 49).

**Table 4:** Multiple regression analysis for VAT and SAT adipose tissue and apparent lipid volume loss.

Dependent variable	*R*²	Independent variables	Coefficient *b*	Std. Error	*t*	*p*
∆ SAT_TV_ (%)	0.32	Constant	−0.42			
		Normalized baseline lower third SAT volume, L/cm	0.24	0.07	3.22	<0.01
		Body fat, %	0.003	0.001	2.41	0.02
		^a^Age, years	–			
		^a^Sex	–			
		^a^Normalized baseline SAT volume, L/cm	–			
		^a^Normalized baseline SAT app. lipid volume, L/cm	–			
		^a^Normalized baseline upper third SAT volume, L/cm	–			
∆ SAT_LV_ (%)	0.38	Constant	−0.47			
		Normalized baseline lower third SAT volume, L/cm	0.28	0.08	3.54	<0.01
		Body fat, %	0.003	0.001	2.76	0.01
		^a^Age, years	–			
		^a^Sex	–			
		^a^Normalized baseline SAT volume, L/cm	–			
		^a^Normalized baseline SAT app. lipid volume, L/cm	–			
		^a^Normalized baseline upper third SAT volume, L/cm	–			
∆ VAT_TV_ (%)	0.23	Constant	−0.35			
		Normalized baseline lower third SAT volume, L/cm	0.32	0.07	4.87	<0.01
		^a^Age, years	–			
		^a^Sex	–			
		^a^Body fat %	–			
		^a^Normalized baseline SAT volume, L/cm	–			
		^a^Normalized baseline SAT app. lipid volume, L/cm	–			
		^a^Normalized baseline upper third SAT volume, L/cm	–			
∆ VAT_LV_ (%)	0.32	Constant	−0.44			
		Normalized baseline SAT volume, L/cm	1.03	0.26	3.90	<0.01
		Normalized baseline middle third SAT volume, L/cm	−0.49	0.23	−2.11	0.04
		^a^Age, years	–			
		^a^Sex	–			
		^a^Normalized baseline SAT app. lipid volume, L/cm	–			
		^a^Normalized baseline upper third SAT volume, L/cm	–			
		^a^Normalized baseline lower third SAT volume, L/cm	–			

Method: stepwise, enter variable if *p* < 0.05, remove variable if *p* > 0.1.

*SAT* subcutaneous adipose tissue, *VAT* visceral adipose tissue, *∆ SAT*_*TV*_ *(%)* relative loss of SAT total volume, *∆ SAT*_*LV*_ *(%)* relative loss of SAT apparent lipid volume, *∆ VAT*_*TV*_ *(%)* relative loss of VAT total volume, *∆ VAT*_*LV*_ *(%)* relative loss of VAT apparent lipid volume, *app.* apparent.

^a^Variables not included in the model.

## Discussion

The present analysis shows that both anthropometric and MRI-measured parameters of AT significantly decrease in people with obesity after an 8-week formula-based LCD as weight loss intervention. The absolute volume change ∆ SAT_TV_ (L) was significantly higher than ∆ VAT_TV_ (L). In contrast, relative volume loss (in % to baseline) was statistically different for apparent lipid volumes, with a higher apparent lipid volume loss in VAT compared to SAT. When dividing SAT and VAT into equidistant thirds in the craniocaudal axis from the liver dome to the femoral heads, the largest volume changes were detectable in the lower third for both SAT and VAT. The strongest predictor for nearly all AT- and apparent lipid volume losses was the normalized SAT volume in the lower third at baseline. The best predictor for VAT apparent lipid volume loss was normalized total SAT volume.

Considering the study design, decreases in obesity markers (anthropometric and MRI-based) were expected since participants underwent an 8-week LCD. A decrease in PDFF in SAT in this cohort has been reported before [47]. Potential explanations are the depletion of lipids from the adipocytes leading to a relative PDFF decrease or an increase in tissue hydration due to the metabolic processes associated with weight loss. Correlations of obesity markers with AT hydration and with AT PDFF are known [36, 48]. The observed correlation of smaller baseline SAT PDFF with greater AT loss in the present longitudinal setting adds a new aspect to these relationships: a lower baseline fat fraction in SAT might be beneficial for AT loss success. PDFF is a good approach to measure MR-visible AT lipid content. Given that lipids are primarily found inside the adipocytes, PDFF potentially serves as an indicator of adipocyte fat content, possibly even offering insights into adipocyte size. Nonetheless, confirming this hypothesis in vivo poses a considerable challenge.

Several weight loss intervention studies have shown that the absolute loss of SAT volume is greater than that of VAT [49–51], which is also evident in the current LION study. There was, however, no significant difference between the relative losses of VAT and SAT volume. When evaluating the changes in apparent lipid volumes in SAT and VAT, VAT exhibited a significantly higher relative loss of apparent lipids compared to SAT. Studies in rodent models may provide possible explanations for these findings: under fasting conditions, the responsiveness of genes related to lipid metabolism is more pronounced in VAT than in SAT [52]. Further, the activity of the sympathetic nervous system toward distinct adipose depots is known to be dynamic and potentially hierarchical, with a switch in lipolytic activity from VAT to SAT in the course of a calorie restriction [53]. Thus, in the relatively short follow-up period of 8 weeks, lipids in VAT were potentially the preferred energy source, however, the total volume loss in SAT compared to VAT was much greater.

Changes in AT volume were most prominent in the lower third of the segmented region, i.e. around the pelvis. It should be emphasized that the location of the AT in MRI studies is, to some extent, different from anthropometric measurements due to the participants’ position during the measurements (lying vs. standing position). In MRI studies, AT is distributed more cranially due to the lack of gravity pulling the AT caudally. Thus, comparisons to studies using waist and hip circumferences are difficult. Previous weight-loss studies found larger changes in abdominal AT than in lower body AT (hip, leg, or gluteofemoral) [23, 54–56]. A study including an overfeeding/underfeeding protocol found that the upper body AT returned to pre–weight-gain levels more rapidly than lower body AT during underfeeding [57]. Differences in study populations and methods could partially explain the discrepancies with the present results. Furthermore, it has to be mentioned that there is no clear definition regarding “lower body fat” or “gluteofemoral fat”, and if and how much of the leg is included varies from study to study.

It is well established that the pattern of AT distribution is a strong determinant of AT functioning (e.g., lipolytic function) and that AT deposition differs between females and males [58, 59]. The present results also show this sex dimorphism in accordance with the classic gynoid and android AT distribution [59]. Men lost more AT than women, especially in the middle and lower VAT, which is in line with previous findings [22, 60]. In contrast, general AT loss was pronounced in the lower SAT in both males and females. To the best of our knowledge, this pronounced SAT loss in the lower third for both males and females has not been reported before, presumably because the MRI methodology applied in the present work, dividing the section from liver dome to femoral heads into thirds, has not been used for AT volume measurements. The finding is somewhat surprising, but the results are corroborated by previous findings showing that a higher waist-to-hip ratio (i.e., less gluteal AT) is advantageous for weight loss [17], and that an increased VAT or VAT/total AT ratio (thus less gluteal SAT) is beneficial for the success of a weight loss intervention [20–22].

AT and apparent lipid volume losses were associated with baseline anthropometric parameters (most strongly to body fat %) and with MRI-measured parameters at baseline. The parameters that correlated best (five highest *r*-values) were included in the multiple regression models to narrow down the key predictors for AT loss. The models revealed that the baseline normalized volume of SAT in the lower abdominopelvic third predicts both SAT and VAT loss, with a smaller volume being associated with greater AT loss. The detected correlations of relative BMI- and weight loss with baseline anthropometric parameters in females- lower body fat % and smaller waist circumference correlated with higher losses- contradict previous findings, where a higher waist circumference was associated with success of a lifestyle intervention for weight loss and body fat % showed no association with success [19]. This could be attributed to differences in study design, the limited BMI range (30.0–39.9 kg/m^2^) of the present study, or the fact that the reported findings are limited to females. However, data on these parameters as predictors for weight loss are scarce [16]. Nevertheless, BMI and weight loss consistently show correlations with SAT volume in the lower third in the present analysis.

### Limitations and strengths

Some limitations of this study have to be considered. Firstly, partial volume effects need to be taken into account when interpreting PDFF measurements with MRI, as PDFF cannot differentiate between intracellular water content and non-lipid tissue portions (e.g., from adjacent organs) within a voxel (3 × 3 × 6 mm³). Secondly, the present calculation of apparent lipid volumes does not take MR-invisible components (non-free-water and non-fat fractions) such as water bound to macromolecules into account; thus, the term “apparent” was employed. However, PDFF provides a good approximation for the actual lipid content [45]. Thirdly, there was a time gap between anthropometric (Institute for Nutritional Medicine) and MRI (Institute of Diagnostic and Interventional Radiology) measurements. Thirdly, compared to studies that measured gluteofemoral AT (measured by thigh circumference, hip circumference, or leg AT mass [61]), the present analysis used segmentations as low as the middle of the femoral head. However, due to the lying position of the participants during the MRI-scan, the AT can be expected to be distributed more cranially in contrast to a standing position. Furthermore, there was no control group for the weight loss intervention, so the 8 weeks of the intervention were identical for all study participants. Lastly, different approaches for normalizing AT volumes could be considered, including BMI, height, or body surface area. The length of the segmented region (from the liver dome to the femoral head) was selected as the parameter of choice as it best accounted for differences in physique with regard to the torso and it yielded the clearest results compared to BMI and body surface area (data not shown).

Some strengths of this study should be mentioned. The use of 3D imaging technologies for AT measurements, as has been applied here, eliminates the limitations of single-slice measurements [32]. Furthermore, we used 3D imaging data in an interventional setting with a relatively large sample size compared to other studies [23]. The chemical shift encoding-based fat quantification method used here has the advantage to be relatively fast, allowing for breath-hold scans minimizing motion artifacts. Using this technique, scans of larger body parts or even a whole body scan could be performed. The segmentation of AT depots was achieved through an automated segmentation pipeline based on deep learning methods after [41, 42], leading to high accuracy and independence from different readers [44]. Moreover, employing this approach results in a noteworthy decrease in segmentation time, surpassing the efficiency of manual or semi-manual segmentation methodologies, as semi-manual segmentation of similar datasets in an earlier study [36] took around 25 min per case. Lastly, the weight loss intervention was highly standardized, increasing the comparability of the results between participants.

## Conclusion

In conclusion, a sex- and depot-specific decrease in AT in people with obesity undergoing an 8-week LCD, measured by anthropometrics and MRI was observed. Results indicated a greater reduction of SAT in the lower third of the abdominopelvic region. The baseline normalized SAT volume in the lower third of the abdominopelvic region predicted both SAT and VAT volume loss, where a smaller volume was associated with greater AT loss. Consequently, measuring SAT volume in the lower abdominopelvic third might help to predict the success of short-term AT loss after an LCD as a weight loss intervention. However, with regard to long-term success and weight cycling, data collected after 12 months will have to be evaluated, and further studies beyond 12 months duration might be necessary.

### Supplementary information

Supplemental Material

## Data Availability

The network results from the deep learning-based automated segmentation pipeline are available at: https://github.com/BMRRgroup/lion-abd-seg-nnunet and https://github.com/BMRRgroup/lion-abd-seg-3dunet. The anthropometric and imaging datasets analyzed during the current study are available from the corresponding author on reasonable request.
