# Supplementary material for: Impact of baseline adipose tissue characteristics on change in adipose tissue volume during a low calorie diet in people with obesity—results from the LION study
Source: Int J Obes (Lond). 2024 Jun 26;48(9):1332–41. doi: 10.1038/s41366-024-01568-6 (PMC11347377; doi:10.1038/s41366-024-01568-6)

## Table S1: MRI sequence parameters for 6-echo chemical shift encoding-based fat quantification method. MRI: magnetic resonance imaging, TR: repetition time, TE: echo time, SENSE: sensitivity encoding for accelerated MRI.

| TR | 7 ms |
| --- | --- |
| TE1/ΔTE | TE1 = 1.14 ms / ΔTE = 0.8 ms |
| Flip angle | 3° |
| Bandwidth | 2367Hz / pixel |
| Acquisition matrix size | 132 x 180 x 19 |
| Field of view (FOV) | 400 x 543 x 144 mm^3^ |
| Acquisition voxel size | 3 x 3 x 6 mm^3^ |
| Acceleration Factor R | 3.5 |
| Reconstructed using Compressed SENSE | |

## **Table S2:** Characteristics of the male (n=32) and female (n=49) study population at baseline and follow-up. BMI: body mass index, SD: standard deviation.

|  | **Parameter** | **Baseline**  **Mean (SD, range)** | **Follow-Up**  **Mean (SD, range)** | **Difference baseline vs. follow-up** |
| --- | --- | --- | --- | --- |
| **Male** | Age, years | 42.34 (10.91, 21 - 58) | 42.54 (10.9, 21 - 58) | -* |
|  | Height, m | 1.81 (0.08, 1.65 - 1.98) | 1.81 (0.08, 1.65 - 1.98) | -* |
|  | Weight, kg | 113.80 (13.20, 85.10 - 149.80) | 100.54 (12.15, 80.70 - 132.20) | P < 0.01 |
|  | BMI, kg/m^2^ | 34.55 (2.96, 30.60 - 39.70) | 30.52 (2.66, 26.80 - 36.00) | P < 0.01 |
|  | Waist circumference, cm | 113.06 (9.22, 95.50 - 135.20) | 101.20 (8.45, 83.60 - 118.50) | P < 0.01 |
|  | Hip circumference, cm | 116.73 (8.55, 102.40 - 136.00) | 109.10 (7.90, 97.30 - 128.40) | P < 0.01 |
|  | Waist-to-hip-ratio WHR | 0.97 (0.07, 0.80 - 1.12) | 0.93 (0.07, 0.76 - 1.05) | P < 0.01 |
|  | Body Fat, % | 31.02 (3.61, 24.80 - 36.90) | 34.53 (7.98, 18.90 - 51.00) | P < 0.01 |
|  |  |  |  |  |
| **Female** | Age, years | 48.88 (10.31, 26 - 65) | 49.04 (10.3, 26 - 66) | -* |
|  | Height, m | 1.66 (0.07, 1.49 - 1.83) | 1.66 (0.07, 1.49 - 1.83) | -* |
|  | Weight, kg | 93.57 (10.29, 71.30 - 124.50) | 83.04 (9.84, 63.40 - 116.00) | P < 0.01 |
|  | BMI, kg/m^2^ | 33.77 (2.59, 30.20 - 39.70) | 29.96 (2.60, 26.10 - 36.00) | P < 0.01 |
|  | Waist circumference, cm | 100.98 (10.40, 76.00 - 126.60) | 93.10 (10.06, 77.70 - 119.20) | P < 0.01 |
|  | Hip circumference, cm | 118.84 (7.72, 101.00 - 135.10) | 111.21 (6.66, 99 - 126.50) | P < 0.01 |
|  | Waist-to-hip-ratio WHR | 0.85 (0.07, 0.72 - 0.98) | 0.84 (0.07, 0.71 - 0.98) | P = 0.08 |
|  | Body Fat, % | 43.57 (3.31, 36.30 - 52.30) | 39.80 (4.49, 28.90 - 51.00) | P < 0.01 |

* not changing with intervention

## Table S3: Adipose tissue characteristics in males (n=32) based on MRI at baseline and follow-up. MRI: magnetic resonance imaging, SAT: subcutaneous adipose tissue, VAT: visceral adipose tissue, PDFF: proton density fat fraction, SD: standard deviation, app.: apparent.

|  | **Parameter** | **Baseline**  **Mean (SD, range)** | **Follow-Up**  **Mean (SD, range)** | **Difference baseline vs. follow-up** |
| --- | --- | --- | --- | --- |
| **SAT** | PDFF, % | 89.78 (1.47, 86.18 - 91.75) | 87.08 (2.75, 80.61 - 90.68) | P < 0.01 |
|  | Total volume, L  Upper third  Middle third  Lower third | 15.11 (4.90, 7.47 - 24.93)  3.76 (1.18, 1.43 - 5.94)  5.35 (1.90, 2.49 - 8.98)  6.01 (2.00, 2.87 - 10.48) | 11.62 (4.29, 5.37 - 20.27)  2.87 (1.07, 1.31 - 4.93)  4.26 (1.65, 1.95 - 7.22)  4.49 (1.71, 1.91 - 8.40) | P < 0.01  P < 0.01  P < 0.01  P < 0.01 |
|  | Normalized volume*, L/cm  Upper third  Middle third  Lower third | 0.32 (0.10, 0.18 - 0.52)  0.24 (0.07, 0.12 - 0.36)  0.35 (0.12, 0.18 - 0.56)  0.39 (0.12, 0.20 - 0.66) | 0.26 (0.09, 0.13 - 0.44)  0.19 (0.06, 0.09 - 0.30)  0.28 (0.10, 0.13 - 0.47)  0.30 (0.10, 0.14 - 0.54) | P < 0.01  P < 0.01  P < 0.01  P < 0.01 |
|  | App. lipid volume, L | 13.62 (4.55, 6.44 - 22.77) | 10.21 (3.97, 4.38 - 18.20) | P < 0.01 |
|  | Normalized app. lipid volume*, L/cm | 0.29 (0.09, 0.15 - 0.48) | 0.22 (0.08, 0.10 - 0.39) | P < 0.01 |
|  |  |  |  |  |
| **VAT** | PDFF, % | 81.31 (2.55, 74.15 - 85.66) | 76.68 (3.98, 67.41 - 81.94) | P < 0.01 |
|  | Total volume, L  Upper third  Middle third  Lower third | 7.51 (2.20, 3.38 - 12.93)  1.66 (0.61, 0.66 - 3.41)  3.96 (1.23, 1.76 - 6.75)  1.90 (0.59, 0.95 - 3.97) | 5.76 (1.82, 2.51 - 9.81)  1.42 (0.56, 0.51 - 2.98)  2.93 (1.00, 1.24 - 4.96)  1.41 (0.42, 0.70 - 2.44) | P < 0.01  P < 0.01  P < 0.01  P < 0.01 |
|  | Normalized volume*, L/cm  Upper third  Middle third  Lower third | 0.16 (0.05, 0.07 - 0.28)  0.11 (0.04, 0.04 - 0.20)  0.26 (0.08, 0.11 - 0.44)  0.12 (0.04, 0.06 - 0.26) | 0.13 (0.04, 0.06 - 0.22)  0.09 (0.04, 0.03 - 0.17)  0.20 (0.06, 0.08 - 0.33)  0.09 (0.03, 0.05 - 0.16) | P < 0.01  P < 0.01  P < 0.01  P < 0.01 |
|  | App. lipid volume, L | 6.15 (1.95, 2.50 - 11.08) | 4.47 (1.57, 1.71 - 8.04) | P < 0.01 |
|  | Normalized app. lipid volume*, L/cm | 0.13 (0.04, 0.05 - 0.24) | 0.10 (0.03, 0.04 - 0.18) | P < 0.01 |
|  |  |  |  |  |
|  | VAT/SAT ratio | 0.55 (0.22, 0.27 - 1.03) | 0.55 (0.22, 0.28 - 1.07) | P = 1 |

* Normalized by the length of the abdominopelvic region in cm

## Table S4: Adipose tissue characteristics in females (n=49) based on MRI at baseline and follow-up. MRI: magnetic resonance imaging, SAT: subcutaneous adipose tissue, VAT: visceral adipose tissue, PDFF: proton density fat fraction, SD: standard deviation, app.: apparent.

|  | **Parameter** | **Baseline**  **Mean (SD, range)** | **Follow-Up**  **Mean (SD, range)** | **Difference baseline vs. follow-up** |
| --- | --- | --- | --- | --- |
| **SAT** | PDFF, % | 90.79 (1.07, 87.54 - 92.61) | 89.32 (1.59, 84.63 - 92.20) | P < 0.01 |
|  | Total volume, L  Upper third  Middle third  Lower third | 15.84 (3.23, 10.21 - 22.05)  4.21 (1.16, 1.44 - 6.89)  5.18 (1.20, 2.89 - 8.02)  6.45 (1.27, 3.52 - 8.91) | 12.77 (3.07, 7.87 - 20.10)  3.37 (1.08, 1.59 - 5.92)  4.23 (1.12, 2.19 - 7.16)  5.16 (1.25, 1.86 - 7.48) | P < 0.01  P < 0.01  P < 0.01  P < 0.01 |
|  | Normalized volume*, L/cm  Upper third  Middle third  Lower third | 0.37 (0.07, 0.25 - 0.60)  0.29 (0.08, 0.11 - 0.53)  0.36 (0.08, 0.21 - 0.58)  0.45 (0.09, 0.24 - 0.68) | 0.30 (0.07, 0.18 - 0.51)  0.24 (0.08, 0.12 - 0.45)  0.30 (0.08, 0.16 - 0.53)  0.37 (0.09, 0.12 - 0.59) | P < 0.01  P < 0.01  P < 0.01  P < 0.01 |
|  | App. lipid volume, L | 14.40 (3.02, 9.20 - 20.34) | 11.44 (2.89, 6.75 - 18.52) | P < 0.01 |
|  | Normalized app. lipid volume*, L/cm | 0.33 (0.07, 0.22 - 0.55) | 0.27 (0.07, 0.15 - 0.46) | P < 0.01 |
|  |  |  |  |  |
| **VAT** | PDFF, % | 77.24 (4.14, 67.43 - 82.80) | 73.36 (4.93, 62.18 - 80.23) | P < 0.01 |
|  | Total volume, L  Upper third  Middle third  Lower third | 4.52 (1.50, 1.91 - 8.47)  0.77 (0.35, 0.25 - 1.62)  2.25 (0.79, 0.79 - 4.39)  1.50 (0.49, 0.70 - 2.82) | 3.61 (1.30, 1.56 - 6.97)  0.68 (0.37, 0.12 - 1.76)  1.75 (0.66, 0.65 - 3.11)  1.18 (0.41, 0.60 - 2.44) | P < 0.01  P = 0.02  P < 0.01  P < 0.01 |
|  | Normalized volume*, L/cm  Upper third  Middle third  Lower third | 0.10 (0.03, 0.04 - 0.18)  0.05 (0.02, 0.02 - 0.11)  0.16 (0.05, 0.05 - 0.27)  0.10 (0.03, 0.04 - 0.18) | 0.09 (0.03, 0.04 - 0.16)  0.05 (0.03, 0.01-0.12)  0.13 (0.05, 0.05 - 0.21)  0.08 (0.03, 0.05 - 0.17) | P < 0.01  P = 0.04  P < 0.01  P < 0.01 |
|  | App. lipid volume, L | 3.54 (1.31, 1.29 - 6.89) | 2.7 (1.11, 0.97 - 5.59) | P < 0.01 |
|  | Normalized app. lipid volume*, L/cm | 0.08 (0.03, 0.03 - 0.14) | 0.06 (0.03, 0.02 - 0.13) | P < 0.01 |
|  |  |  |  |  |
|  | VAT/SAT ratio | 0.29 (0.09, 0.12 - 0.52) | 0.29 (0.09, 0.13 - 0.52) | P = 0.42 |

* Normalized by the length of the abdominopelvic region in cm

## Table S5: Pearson correlation coefficients for VAT and SAT adipose tissue and apparent lipid volume changes in males (n=32) with anthropometric and imaging parameters at baseline. Significance marked as * for p<0.05 and ** for p<0.01. SAT: subcutaneous adipose tissue, VAT: visceral adipose tissue, ∆ SAT_TV_ (%): relative loss of SAT total volume, ∆ SAT_LV_ (%): relative loss of SAT apparent lipid volume, ∆ VAT_TV_ (%): relative loss of VAT total volume, ∆ VAT_LV_ (%): relative loss of VAT apparent lipid volume, app.: apparent.

| **Parameters at baseline** | | **∆ SAT_TV_ (%)** | **∆ SAT_LV_ (%)** | **∆ VAT_TV_ (%)** | **∆ VAT_LV_ (%)** |
| --- | --- | --- | --- | --- | --- |
| **Anthropometry** | BMI, kg/m² | 0.38* | 0.40* | 0.35* | 0.40* |
|  | Body Fat, % | 0.46** | 0.48** | 0.49** | 0.51** |
|  | Waist circumference, cm | 0.39* | 0.42* | 0.31 | 0.37* |
|  | Hip circumference, cm | 0.38* | 0.42* | 0.39* | 0.42* |
|  | Waist-Hip-Ratio | 0.07 | 0.07 | -0.01 | 0.02 |
| **SAT** | PDFF, % | 0.25 | 0.29 | 0.42* | 0.43* |
|  | Normalized volume^†^, L/cm   - Total - Upper - Middle - Lower | 0.45**  0.38*  0.40*  0.51** | 0.51**  0.44*  0.45**  0.55** | 0.51**  0.44*  0.42*  0.58** | 0.55**  0.50**  0.46**  0.62** |
|  | Normalized app. lipid volume^†^, L/cm | 0.45* | 0.50** | 0.50** | 0.55** |
| **VAT** | PDFF, % | 0.37* | 0.37* | 0.35* | 0.37* |
|  | Normalized volume^†^, L/cm   - Total - Upper - Middle - Lower | 0.32  0.38*  0.28  0.31 | 0.33  0.39*  0.28  0.32 | 0.20  0.18  0.17  0.25 | 0.23  0.22  0.20  0.28 |
|  | Normalized app. lipid volume^†^, L/cm | 0.34 | 0.34 | 0.21 | 0.25 |
|  | VAT/SAT ratio | -0.11 | -0.15 | -0.22* | -0.23* |

^†^ Normalized by the length of the abdominopelvic region in cm

## Table S6: Pearson correlation coefficients for VAT and SAT adipose tissue and apparent lipid volume changes in females (n=49) with anthropometric and imaging parameters at baseline. Significance marked as * for p<0.05 and ** for p<0.01. SAT: subcutaneous adipose tissue, VAT: visceral adipose tissue, ∆ SAT_TV_ (%): relative loss of SAT total volume, ∆ SAT_LV_ (%): relative loss of SAT apparent lipid volume, ∆ VAT_TV_ (%): relative loss of VAT total volume, ∆ VAT_LV_ (%): relative loss of VAT apparent lipid volume, app.: apparent.

| **Parameters at baseline** | | **∆ SAT_TV_ (%)** | **∆ SAT_LV_ (%)** | **∆ VAT_TV_ (%)** | **∆ VAT_LV_ (%)** |
| --- | --- | --- | --- | --- | --- |
| **Anthropometry** | BMI, kg/m² | 0.40** | 0.41** | 0.32* | 0.37** |
|  | Body Fat, % | 0.45** | 0.50** | 0.39** | 0.48** |
|  | Waist circumference, cm | 0.27 | 0.30* | 0.41** | 0.44** |
|  | Hip circumference, cm | 0.30* | 0.33* | 0.17 | 0.20 |
|  | Waist-Hip-Ratio | 0.09 | 0.10 | 0.37** | 0.38** |
| **SAT** | PDFF, % | 0.25 | 0.26 | 0.26 | 0.34* |
|  | Normalized volume^†^, L/cm   - Total - Upper - Middle - Lower | 0.40**  0.28  0.31*  0.44** | 0.44**  0.31*  0.34*  0.47** | 0.39**  0.36*  0.35*  0.32* | 0.46**  0.44**  0.40**  0.37** |
|  | Normalized app. lipid volume^†^, L/cm | 0.39** | 0.43** | 0.38** | 0.45** |
| **VAT** | PDFF, % | 0.21 | 0.23 | 0.20 | 0.26 |
|  | Normalized volume^†^, L/cm   - Total - Upper - Middle - Lower | 0.27  0.13  0.25  0.30* | 0.29*  0.14  0.27  0.33* | 0.20  0.02  0.22  0.25 | 0.27  0.07  0.29*  0.33* |
|  | Normalized app. lipid volume^†^, L/cm | 0.26 | 0.28* | 0.20 | 0.28 |
|  | VAT/SAT ratio | 0.02 | 0.02 | -0.03 | 0.002 |

^†^ Normalized by the length of the abdominopelvic region in cm

## **Figure S1:** Dot plots showing comparisons of SAT and VAT volume losses after the 8-week weight loss intervention. Horizontal lines represent the mean with its 95% confidence interval. A) Absolute loss of SAT and VAT volume and SAT apparent lipid and VAT apparent lipid volume. SAT loss was significantly larger for both total AT volume and for apparent lipid volume (p < 0.01). B) Loss of SAT and VAT volume and SAT apparent lipid and VAT apparent lipid volume relative to baseline. In relative terms, only SAT lipid loss was larger than VAT lipid loss (p < 0.01). SAT: subcutaneous adipose tissue, VAT: visceral adipose tissue, AT: adipose tissue, ∆ SAT_TV_ [L]: absolute loss of SAT total volume, ∆ SAT_LV_ [L]: absolute loss of SAT apparent lipid volume, ∆ VAT_TV_ [L]: absolute loss of VAT total volume, ∆ VAT_LV_ [L]: absolute loss of VAT apparent lipid volume ∆ SAT_TV_ [%]: relative loss of SAT total volume, ∆ SAT_LV_ [%]: relative loss of SAT apparent lipid volume, ∆ VAT_TV_ [%]: relative loss of VAT total volume, ∆ VAT_LV_ [%]: relative loss of VAT apparent lipid volume.


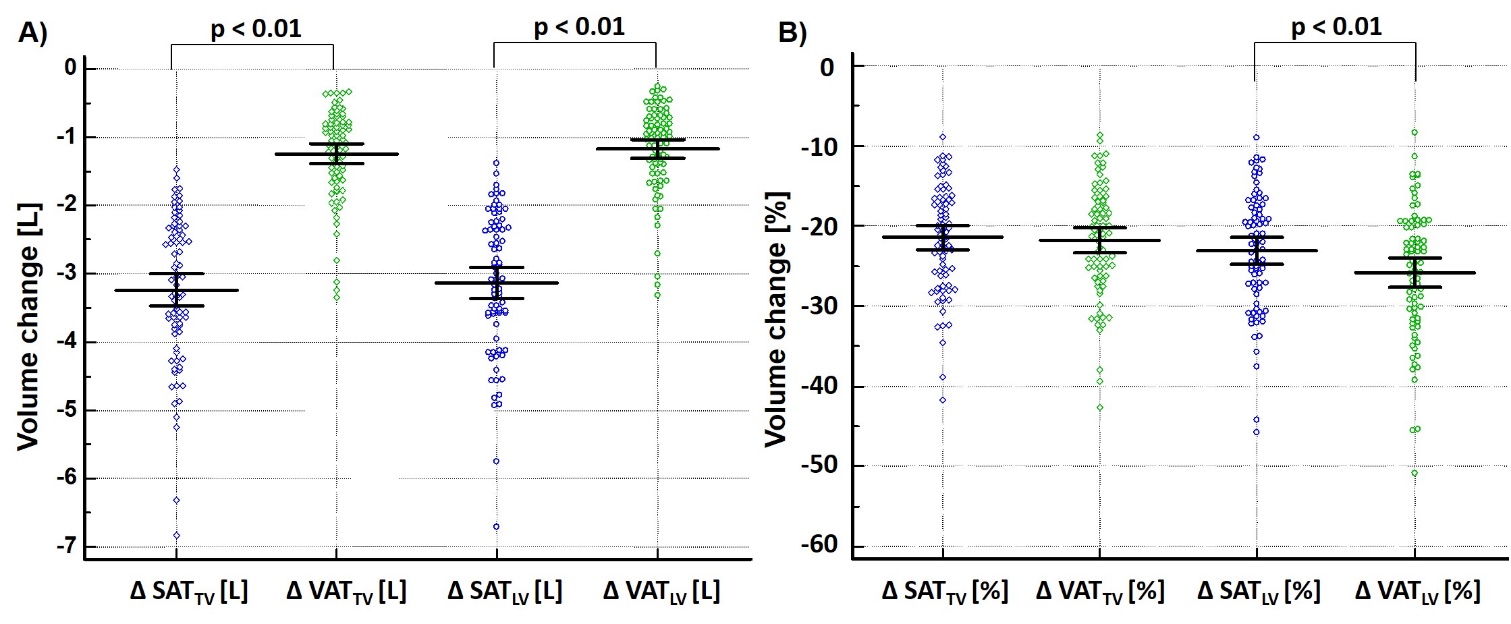


## **Figure S2**: Dot plots showing comparisons of relative SAT and VAT volume losses as well as SAT apparent lipid and VAT apparent lipid volume losses after the 8-week weight loss intervention in females (left) and males (right). Horizontal lines represent the mean with its 95% confidence interval. Relative to baseline, only the loss of lipids was larger in SAT than in VAT, both in females (p < 0.01) and in males (p = 0.03). SAT: subcutaneous adipose tissue, VAT: visceral adipose tissue, ∆ SAT_TV_ [%]: relative loss of SAT total volume, ∆ SAT_LV_ [%]: relative loss of SAT apparent lipid volume, ∆ VAT_TV_ [%]: relative loss of VAT total volume, ∆ VAT_LV_ [%]: relative loss of VAT apparent lipid volume.


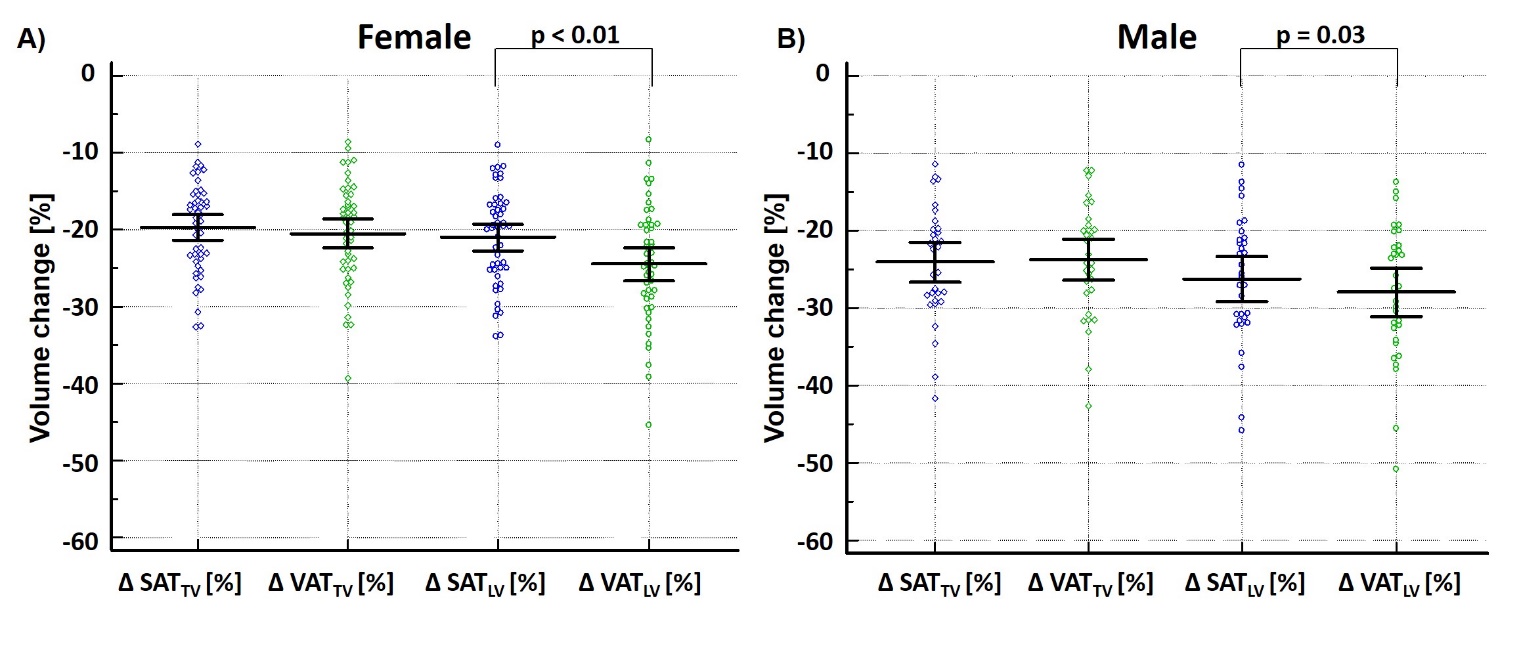

Supplement: Supplementary file 1 — Supplemental Material [file 41366_2024_1568_MOESM1_ESM.docx]
